# Exosomes from Microvascular Endothelial Cells under Mechanical Unloading Inhibit Osteogenic Differentiation via miR-92b-3p/ELK4 Axis

**DOI:** 10.3390/jpm12122030

**Published:** 2022-12-08

**Authors:** Xiaoyan Zhang, Lijun Zhang, Liqun Xu, Gaozhi Li, Ke Wang, Tong Xue, Quan Sun, Hao Tang, Xinsheng Cao, Zebing Hu, Shu Zhang, Fei Shi

**Affiliations:** The Key Laboratory of Aerospace Medicine, Ministry of Education, Air Force Medical University, Xi’an 710032, China

**Keywords:** mechanical unloading, exosomes, miR-92b-3p, osteogenic differentiation

## Abstract

Mechanical unloading-related bone loss adversely harms astronauts’ health. Nevertheless, the specific molecular basis underlying the phenomenon has not been completely elucidated. Although the bone microvasculature contributes significantly to bone homeostasis, the pathophysiological role of microvascular endothelial cells (MVECs) in bone loss induced by mechanical unloading is not apparent. Here, we discovered that MC3T3-E1 cells could take up exosomes produced by MVECs under clinorotation-unloading conditions (Clino Exos), which then prevented MC3T3-E1 cells from differentiating into mature osteoblasts. Moreover, miR-92b-3p was found to be highly expressed in both unloaded MVECs and derived exosomes. Further experiments demonstrated that miR-92b-3p was transferred into MC3T3-E1 cells by exosomes, resulting in the suppression of osteogenic differentiation, and that encapsulating miR-92b-3p inhibitor into the Clino Exos blocked their inhibitory effects. Furthermore, miR-92b-3p targeted ELK4 and the expression of ELK4 was lessened when cocultured with Clino Exos. The inhibitor-92b-3p-promoted osteoblast differentiation was partially reduced by siRNA-ELK4. Exosomal miR-92b-3p secreted from MVECs under mechanical unloading has been shown for the first time to partially attenuate the function of osteoblasts through downregulation of ELK4, suggesting a potential strategy to protect against the mechanical unloading-induced bone loss and disuse osteoporosis.

## 1. Introduction

Numerous studies have illustrated that mechanical stimuli are crucial for keeping the equilibrium between bone formation and resorption via mechanical transduction pathways [1,2]. However, the weightless environment of spaceflight disrupts bone remodeling and homeostasis, resulting in severe bone loss and even disuse osteoporosis, which has become a major concern for astronauts’ health [3]. During a 4- to 6-month space mission, astronauts lose weight-bearing bone mass at a rate of 0.5% to 1.5% per month, and bone mass fail to return to normal for an extended period of time after the astronaut returns to the ground [4]. Given that concerns about mechanical unloading-induced bone loss severely restrict the advancement of manned spaceflight missions, it is crucial to unravel the molecular mechanism of weightless bone loss and then advance research on critical preventative measures.

Previous research has focused on the mechanisms of functional changes in osteoclasts and osteoblasts and their mutual regulation under mechanical unloading [5,6]. Accumulating evidence has indicated that skeletal capillaries are critical for maintaining bone homeostasis and that there is a strong spatiotemporal link between angiogenesis and bone formation; this link is known as angiogenesis–osteogenesis coupling [7]. In addition, microvascular endothelial cells (MVECs) exert a crucial impact on the coupling process. Research has shown when human mesenchymal stem cells (HMSCs) are cocultured with human dermal microvascular endothelial cells (HDMECs), the osteoblast differentiation of HMSCs is accelerated by elevating the expression of osteogenesis-related genes and alkaline phosphatase (ALP) activity [8]. Ribeiro et al. also suggested that in cocultured HDMECs/HMSCs osteogenic gene expression and ALP activity are greatly induced [9]. Additionally, human osteoblasts were cultured using conditioned media obtained from human microvascular endothelial cells in mechanical unloading, and it was discovered that this medium impeded osteoblast proliferation and inhibited osteoblast activity [10]. These results suggest that mechanical unloading may indirectly interfere with osteoblast function by impacting the intercellular communication between MVECs and osteoblasts.

For the past few years, exosomes, a new pathway for intercellular communication, have received much attention from researchers [11]. Exosomes produced by almost all kinds of cells are vesicles with phospholipid bilayers of 40–160 nm in diameter [12]. Research has shown that exosomes play regulatory roles through delivering nucleic acids, proteins, or other regulatory factors into receptor cells and participate in various physiological activities, such as the immune response, bone reconstruction, and angiogenesis [11,12]. microRNAs (miRNAs), a crucial component of exosomes, are noncoding RNAs consisting of approximately 22 nucleotides [12,13]. Numerous research reports have suggested that miRNAs modulate a wide range of biological processes through binding with target mRNAs in the 3′-untranslated regions (UTRs) to regulate gene expression at the posttranscriptional phase [13]. A vast variety of miRNAs, including miR-132-3p, miR-139-3p, miR-320-3p, and others, have been discovered to influence osteoblast activity in mechanical unloading [14,15,16]. In the bone microenvironment, miRNAs can be delivered by exosomes produced by several cell types, such as bone marrow mesenchymal stem cells (BMSCs), osteoblasts, and osteoclasts [17,18,19,20]. However, it has not been reported whether MVECs under mechanical unloading can regulate osteoblast function through exosomal delivery of miRNAs.

In this article, we reported that exosomes produced by MVECs cultured under mechanical unloading retarded the osteogenic differentiation. Through analysis of RNA sequencing data and unloading studies, miR-92b-3p was identified as the miRNA with the most noticeably increased expression. Our study further revealed that miR-92b-3p, which was transferred by exosomes of MVECs cultured under mechanical unloading, specifically targeted ELK4, causing osteoblast differentiation to be hampered. This work will help deepen the understanding of the mechanism of bone loss under weightlessness and provide novel strategies for protection against and therapy of weightless bone loss.

## 2. Materials and Methods

### 2.1. Cell Culture and In Vitro Differentiation

The Cell Bank of the Chinese Academy of Sciences (Shanghai, China) provided the mouse preosteoblastic cell line MC3T3-E1 and the mouse microvascular endothelial cell line bEnd.3. MVECs were grown in Dulbecco’s Modified Eagle Medium (DMEM) with high glucose containing 1% penicillin/streptomycin (Gibco, New York, NY, USA) and 10% FBS (Gibco, USA). MC3T3-E1 cells were grown in alpha-Minimum Essential Medium (α-MEM) containing 100 units/mL penicillin/streptomycin (Gibco, USA), and 10% FBS (Gibco, USA). Osteogenic media adding 100 nM dexamethasone, 10 mM -glycerophosphate (Sigma, Burlington, MA, USA), and 50 μM ascorbic acid was used to culture MC3T3-E1 cells until they reached the appropriate confluency for the osteogenic differentiation experiments. Both types of cells were cultivated in a cell incubator with 95% humidity and 5% CO_2_ at 37 °C. Cells beyond passage 12 were not used. All cell studies were performed 3 times (*n* = 3).

### 2.2. Two-Dimensional Clinorotation

The environment of microgravity in cells on the ground is frequently simulated using 2D clinorotation. The process of related experiments was explained in detail previously [21]. MVECs were routinely cultured after being seeded at a proper concentration in a specific cell culture flask. After cells reached 40% confluency, the culture flasks were entirely loaded with medium containing 10% exosome-depleted FBS (VivaCell Biosciences, Shanghai, China) and plugged, guaranteeing that there were no air bubbles. Following fixation in the 2D clinostat, the culture flasks were rotated for 48 h around the horizontal axis. The control groups were not rotated and were kept in the same environment.

### 2.3. Exosome Isolation and Characterization

MVECs were grown in exosome-free medium. After clinorotation for 48 h, exosomes were extracted via ultracentrifugation from cell supernatants. Briefly, the harvested culture supernatant was centrifuged at 300× *g* for 10 min at 4 °C, then again at 2000× *g* for 10 min, and finally at 10,000× *g* for 30 min to eliminate cellular debris and dead cells from the medium. Then, the collected supernatant was ultracentrifuged (Beckman Coulter, Brea, CA, USA) at 100,000× *g* for 70 min. To remove contaminating proteins, the exosomes were washed with PBS and subsequently ultracentrifuged at 100,000× *g* for another 70 min. After carefully removing the supernatant, the pellets were resuspended in PBS and kept at −80 °C until use. The protein concentration in the exosome suspension was detected by a Pierce™ BCA Protein Assay Kit (Thermo Fisher Scientific, Waltham, MA, USA). Flow NanoAnalyzer (NanoFCM, Xiamen, China) was implemented to evaluate the size distribution of isolated exosomes. TEM (Hitachi, Tokyo, Japan) was applied to examine the morphology of the extracted exosomes.

### 2.4. Exosome Internalization

Following a 30 s vortex, exosomes were stained with DiI (Beyotime Biotechnology, Shanghai, China) by incubating them for 30 min at 37 °C with the dye at a ratio of 500:1 in a dark room. Next, the resulting solution was ultracentrifuged at 100,000× *g* for 70 min, twice, using PBS. The harvested exosomes were diluted in 200 μL PBS. Then, MC3T3-E1 cells were cultivated with DiI-stained exosomes for 3 h after a 60 s vortex. After three PBS washes, the MC3T3-E1 cells were incubated with 4% paraformaldehyde for 10 min. The cell nuclei and the cytoskeleton were labeled with DAPI and phalloidin-FITC (Beyotime Biotechnology, China), respectively, according to the manufacturer’s guidelines. Finally, the confocal microscope was utilized to capture images.

### 2.5. miRNA Loading into Exosomes

In 0.4 cm wide electroporation cuvettes, miR-92b-3p inhibitor or negative control (1 OD) (GenePharma, Shanghai, China) was electroporated into exosomes (1 mg/mL) at 700 V/150 mF (BIO-RAD, Hercules, CA, USA). Supplementary Table S2 includes a list of the miRNAs’ sequences. Subsequently, RNase was used to eliminate any miRNAs that might have remained attached to the membrane of exosomes after electroporation. The cold PBS was then used to rinse the exosomes three times with ultracentrifugation at 100,000× *g* for 70 min. The exosome solution was further vortexed to separate any potential aggregates.

### 2.6. qRT–PCR

RNAiso Plus (TaKaRa, Shiga, Japan) was utilized to isolate total RNA and the standard reverse transcription procedure was followed. mRNA was reverse-transcribed using a Prime ScriptTM RT Master Mix Kit (TaKaRa, Japan). Reverse transcription of miRNA into cDNA was accomplished using the Mir-X miRNA First-Strand Synthesis Kit (TaKaRa, Japan). The endogenous controls were GAPDH or U6 small nuclear RNA. CFX96 real-time PCR detection system (BIO-RAD, Hercules, CA, USA) was applied to measure the expression of the target genes using SYBR^®^ Premix Ex Taq TM II (TaKaRa, Japan). In Supplementary Table S1, the primer sequences are displayed.

### 2.7. Western Blotting Analysis

Both types of cells were lysed on ice with M-PER Mammalian Protein Extraction Reagent (Thermo Fisher Scientific, USA) supplemented with 10% protease inhibitor cocktail (Roche, Mannheim, Germany) to isolate total protein and then measured by a Pierce™ BCA Protein Assay Kit (Thermo Fisher Scientific, USA) in accordance with the manufacturer’s instructions. Protein samples supplemented with loading buffer in equal proportions were electrophoresed on NuPAGE™ Bis-Tris Protein Gels (Invitrogen, Waltham, MA, USA) and then transferred onto polyvinylidene fluoride (PVDF) membranes. After incubation with 5% skim milk (5% *w*/*v*) for 2 h at room temperature, the membranes were co-incubated overnight at 4 °C with the following primary antibodies specific for GAPDH (1:1000; Cell Signaling Technology, USA), Runx2 (1:1000; Cell Signaling Technology, Danvers, MA, USA), Osx (1:1000; Abcam, Cambridge, UK), Ocn (1:2000; Abcam, UK), ELK4 (1:1000; Proteintech, Rosemont, IL, USA), GM130 (1:1000; Proteintech, USA), CD9 (1:1000; Proteintech, USA), and TSG101 (1:1000; Proteintech, USA). Next, the peroxidase-conjugated secondary antibody (1:5000; ZS-GB-BIO, Beijing, China) was utilized to treat the PVDF membranes for 2 h at room temperature. The ECL kit (Thermo Fisher Scientific, USA) was employed to visualize the signals. Densitometry analysis was conducted with ImageJ software.

### 2.8. Cell Transfection

The miR-92b-3p mimic (40 nM), miR-92b-3p inhibitor (80 nM), siRNA-ELK4 (80 nM), and their respective negative controls (GenePharma, China) were transfected into MC3T3-E1 cells by using Lipofectamine 2000 (Invitrogen, USA) in accordance with the manufacturer’s instructions. The sequences of the siRNAs and negative controls specific for miR-92b-3p and ELK4 are displayed in Supplementary Table S2.

### 2.9. ALP Activity Assay

M-PER Mammalian Protein Extraction Reagent (Thermo Fisher Scientific, USA) was applied to isolate total protein from MC3T3-E1 cells. The concentration of protein was determined utilizing a Pierce™ BCA Protein Assay Kit (Thermo Fisher Scientific, USA). ALP activity was assessed by an ALP assay kit (Nanjing Jiancheng Technological, Nanjing, China). The amount of phenol produced following a 15 min reaction of 1 g protein with the substrate at 37 °C was applied to evaluate ALP activity (IU/L).

### 2.10. ALP Staining

ALP staining of MC3T3-E1 cells that had been induced for 7 days in osteogenic media was performed with the BCIP/NBT staining kit (Beyotime Biotechnology, China) following the manufacturer’s instructions. A digital camera was used to capture representative images.

### 2.11. Luciferase Assay

Utilizing the bioinformatics tool TargetScan, miRWalk, and miRDB, we predicted the potential binding sites between miR-92b-3p and the ELK4 3′UTR sequence. The 3′UTR fragment of ELK4 containing miR-92b-3p binding site wild-type (WT) or mutant (MUT) was cloned into a pmir-GLO Dual-luciferase Target Vector (Promega, Madison, WI, USA). The pmir-GLO vector (WT fragments or MUT fragments) and miR-92b-3p mimic or the negative control were transfected into the 293T cells using Lipofectamine 2000 (Invitrogen, USA). The Dual-Luciferase Reporter Assay System (Promega, USA) was implemented to measure luciferase activity 48 h after transfection according to the guidelines. The co-expressed Renilla luciferase activity was used to normalize the firefly luciferase activity.

### 2.12. Statistical Analysis

SPSS 22.0 software was employed to analyze the data, which were displayed as the means ± SDs. All experiments were repeated in triplicate. Comparisons between two groups were performed using Student’s two-sided *t*-test, and multiple-group comparisons were analyzed by one-way ANOVA followed by the LSD multiple comparisons test; *p* < 0.05 was considered statistically significant.

## 3. Results

### 3.1. Isolation, Identification, and Internalization of MVEC-Derived Exosomes

**Figure 1:**
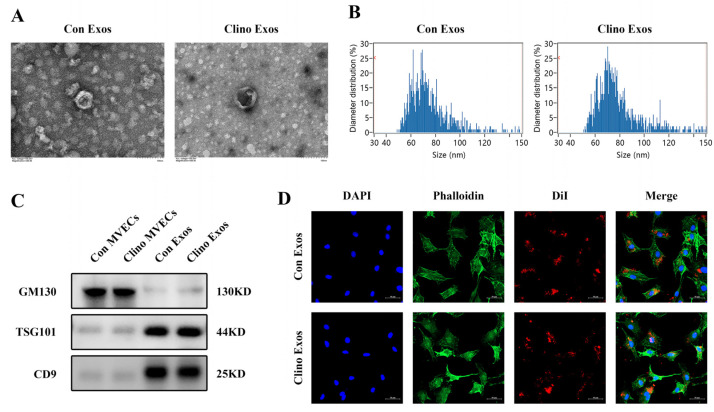
Isolation, identification, and internalization of MVEC-derived exosomes. (**A**) TEM demonstrated the typical characteristics of exosomes generated from MVECs (scale bar, 100 nm). (**B**) Flow NanoAnalyzer analyzed the size distribution of exosomes. (**C**) Exosomal/cellular protein markers in donor cells and extracted exosomes detected by Western blotting analysis. (**D**) Confocal microscopy images displaying the absorption of exosomes secreted from MVECs by MC3T3-E1 cells. DiI-labeled MVEC-derived exosomes are red, and DAPI (blue) and phalloidin (green) labeled the cells (scale bar, 50 μm).

Exosomes produced by MVECs cultured under normal conditions (Con Exos) and with clinorotation (Clino Exos) were isolated through multiple centrifugation procedures. The morphological characteristics of Con Exos and Clino Exos were identified by TEM, which demonstrated the disc-like morphology typical of exosomes (Figure 1A). Flow NanoAnalyzer analysis revealed that the two groups of vesicles were between 50 and 120 nm in diameter (Figure 1B). Western blotting analysis further indicated that these particles all highly expressed TSG101 and CD9, two exosomal markers, but not the cell-specific marker GM130 (Figure 1C). To examine whether MVEC-derived exosomes can be absorbed into MC3T3-E1 cells, two sets of fluorescent dye (DiI)-labeled exosomes were incubated with MC3T3-E1 cells. According to confocal fluorescence microscopy, both groups of exosomes could be absorbed by MC3T3-E1 cells (Figure 1D).

### 3.2. Exosomes Derived from MVECs Cultured under Mechanical Unloading Attenuate Osteoblast Differentiation

**Figure 2:**
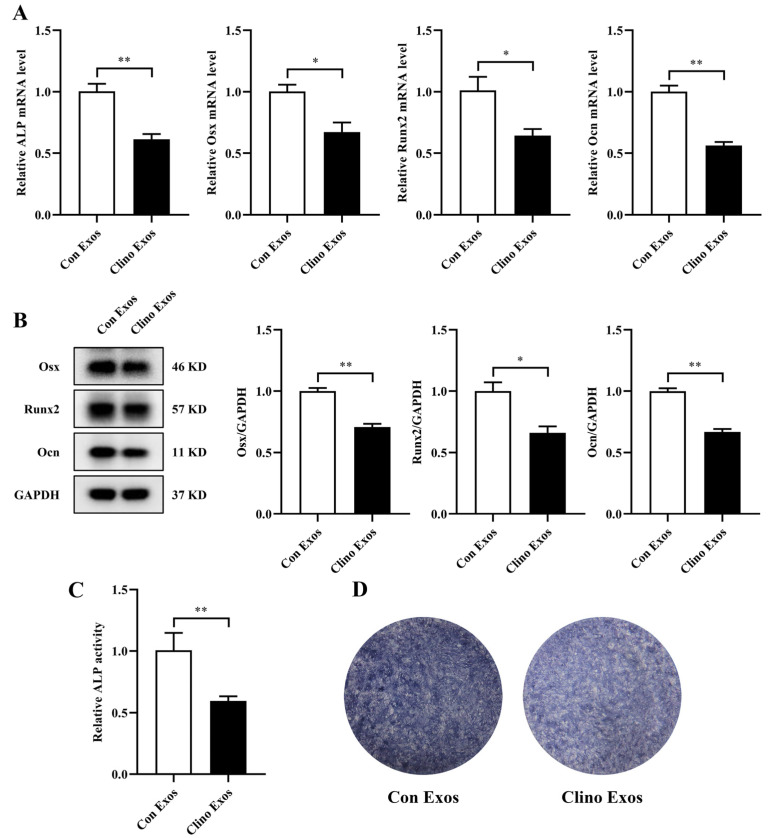
Exosomes derived from MVECs cultured under mechanical unloading attenuate osteoblast differentiation. (**A**) qRT–PCR analysis of ALP, Osx, Runx2, and Ocn in MC3T3-E1 cells treated with Con Exos/Clino Exos (200 μg/mL) (*n* = 3). (**B**) Western blotting analysis of Osx, Runx2, and Ocn in MC3T3-E1 cells treated with Con Exos/Clino Exos (*n* = 3). (**C**) Relative ALP activity analysis of osteoblasts treated with Con Exos/Clino Exos (*n* = 3). (**D**) Representative images of ALP staining in MC3T3-E1 cells treated with Con Exos/Clino Exos after osteoinduction for 7 days (*n* = 3). * *p* < 0.05, ** *p* < 0.01 vs. control.

To further investigate whether Clino Exos were able to affect osteoblast differentiation, Con Exos and Clino Exos were added to MC3T3-E1 cells in culture. The results indicated that Clino Exos significantly decreased the mRNA expression levels of osteoblast-specific markers, including alkaline phosphatase (ALP), Osterix (Osx), Runt-related transcription factor 2 (Runx2), and Osteocalcin (Ocn), compared with the Con Exos group (Figure 2A). In accordance with these results, the protein expression levels of Osx, Runx2, and Ocn were markedly reduced in the Clino Exos treatment group (Figure 2B). ALP activity and ALP staining also exhibited comparable trends (Figure 2C,D). Collectively, these consequences revealed that Clino Exos attenuated the osteoblast differentiation.

### 3.3. miR-92b-3p Is Elevated in MVEC-Derived Exosomes Cultured under Mechanical Unloading

**Figure 3:**
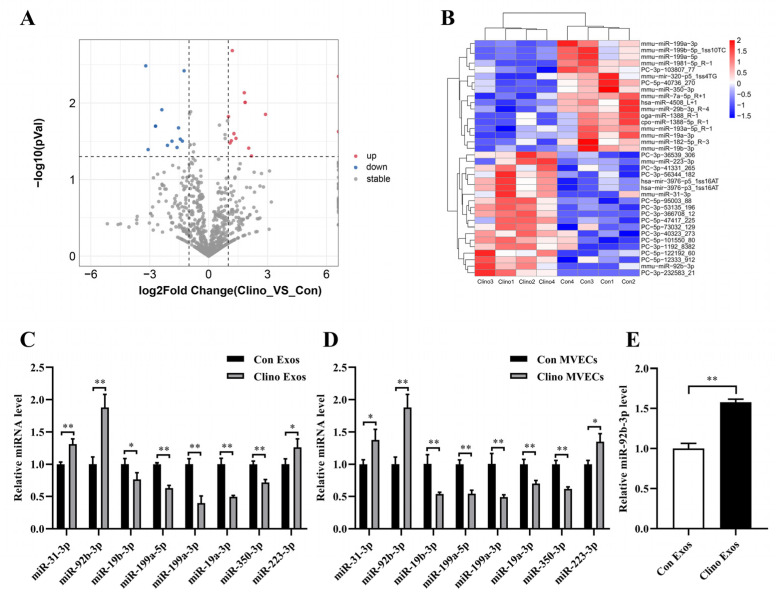
miR-92b-3p is elevated in MVEC-derived exosomes cultured under mechanical unloading. (**A**) Representative volcano plot of different levels of miRNAs between Con Exos and Clino Exos. (**B**) Representative heatmap of different levels of miRNAs between Con Exos and Clino Exos. (**C**) Differential miRNAs expression of Con Exos and Clino Exos was validated by qRT–PCR (*n* = 3). (**D**) qRT–PCR was used to examine the differential expression of miRNAs chosen from RNA sequencing data in MVECs subjected to mechanical unloading (*n* = 3). (**E**) qRT–PCR illustrated the level of miR-92b-3p in MC3T3-E1 cells incubated with Con Exos/Clino Exos (200 μg/mL) (*n* = 3). * *p* < 0.05, ** *p* < 0.01 vs. control.

miRNAs, conserved among species, are the major component of exosomes and perform critical regulatory functions in target cells. Therefore, we carried out small RNA sequencing to examine the small RNAs in Con Exos and Clino Exos to determine which miRNAs were the primary functional mediators in MVEC-generated exosomes. The RNA sequencing results indicated that 19 miRNAs were upregulated and 17 were downregulated in Clino Exos relative to Con Exos (*p* < 0.05) (Figure 3A,B). Then, we validated whether the changes in the eight named miRNAs in Con Exos and Clino Exos were consistent with the small RNA sequencing results and confirmed whether their expression changed in MVECs under clinorotation (Figure 3C,D). Notably, the qRT–PCR verification results showed similar trends (Figure 3C,D). Considering above findings, we selected miR-92b-3p as the candidate miRNA since its expression was noticeably elevated. Furthermore, we performed qRT-PCR analysis to measure the level of miR-92b-3p in MC3T3-E1 cells cultured with Clino Exos and Con Exos. The results showed that MC3T3-E1 cells cultivated with Clino Exos had a notably greater level of miR-92b-3p than cells treated with Con Exos (Figure 3E).

### 3.4. miR-92b-3p Hinders the Osteogenic Differentiation of MC3T3-E1 Cells

**Figure 4:**
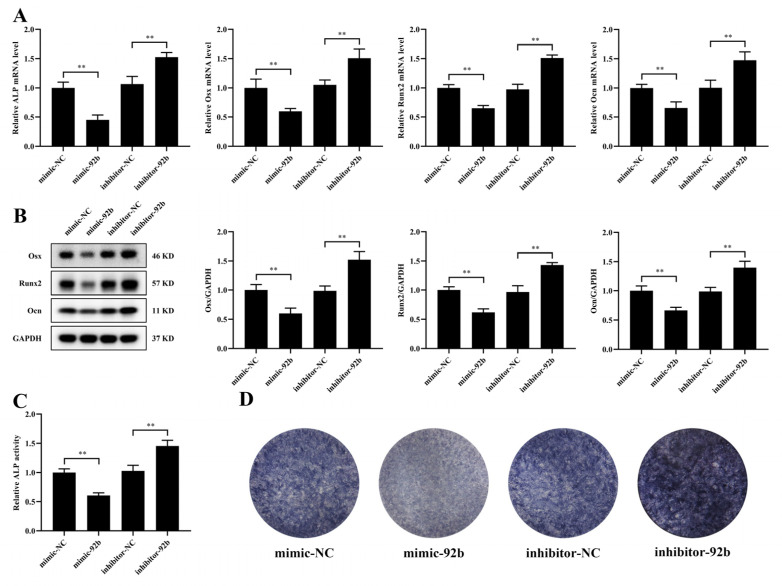
miR-92b-3p hinders the osteogenic differentiation of MC3T3-E1 cells. miR-92b-3p mimic, inhibitor, and miRNA negative controls were transfected into MC3T3-E1 cells. (**A**) mRNA expression of ALP, Osx, Runx2, and Ocn in MC3T3-E1 cells analyzed by qRT–PCR (*n* = 3). (**B**) Protein levels of Osx, Runx2, and Ocn in MC3T3-E1 cells analyzed by Western blotting (*n* = 3). (**C**) Relative ALP activity analysis in MC3T3-E1 cells (*n* = 3). (**D**) Representative staining images for ALP in MC3T3-E1 cells (*n* = 3). ** *p* < 0.01 vs. control.

miR-92b-3p is predominantly regarded as a tumor suppressor or carcinogen in several tumors. However, the precise function of miR-92b-3p in the osteogenic differentiation of MC3T3-E1 cells has not been illustrated. To explore whether miR-92b-3p contributed to the inhibition of osteogenic differentiation, we transfected a miR-92b-3p mimic or inhibitor into MC3T3-E1 cells. Transfection of miR-92b-3p mimic significantly reduced the mRNA and protein expression of Osx, Runx2, and Ocn compared with the NC-mimic, while the miR-92b-3p inhibitor had the opposite effect (Figure 4A,B). Moreover, results consistent with these findings were observed for the mRNA level of ALP, ALP activity, and ALP staining (Figure 4A,C,D). In brief, these consequences showed that miR-92b-3p regulated the osteoblast differentiation negatively.

### 3.5. Exosomes Derived from MVECs Cultured under Mechanical Unloading Inhibit Osteogenic Differentiation by Transferring miR-92b-3p

**Figure 5:**
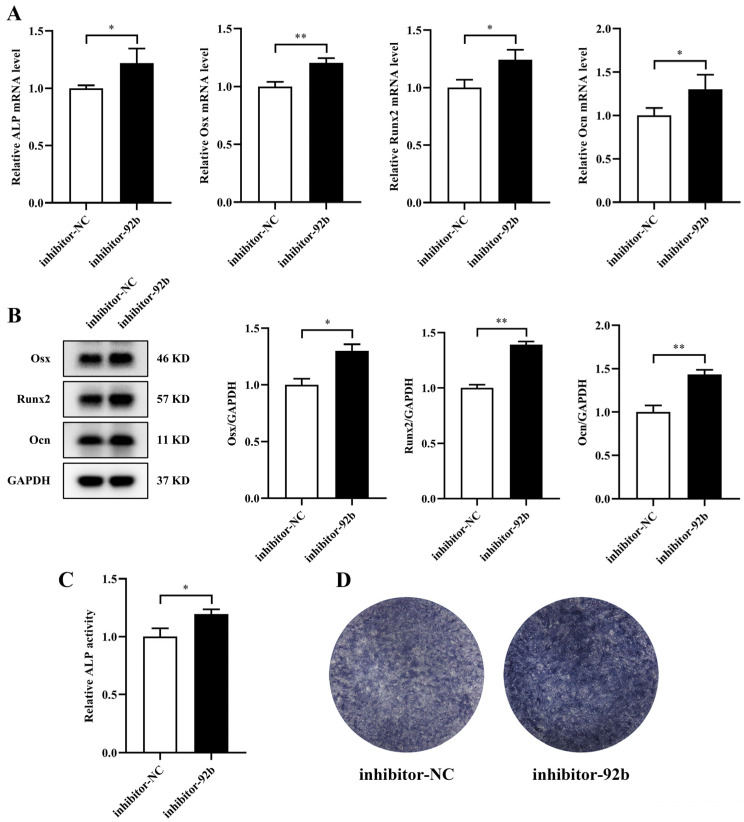
Exosomes derived from MVECs cultured under mechanical unloading inhibit osteogenic differentiation by transferring miR-92b-3p. miR-92b-3p inhibitor and NC-inhibitor were transfected into Clino Exos via electroporation. (**A**) mRNA expression levels of ALP, Osx, Runx2, and Ocn in MC3T3-E1 cells treated with Clino Exos (electroporated with miR-92b-3p inhibitor/NC-inhibitor, 200 μg/mL) (*n* = 3). (**B**) Protein levels of Osx, Runx2, and Ocn in MC3T3-E1 cells treated the same as above (*n* = 3). (**C**) Relative ALP activity analysis in MC3T3-E1 cells treated the same as above (*n* = 3). (**D**) Representative staining images for ALP in MC3T3-E1 cells treated the same as above after 7 days osteogenic induction (*n* = 3). * *p* < 0.05, ** *p* < 0.01 vs. control.

Since miR-92b-3p impeded osteoblast differentiation, we investigated whether Clino Exos could exert negative effects on osteogenic differentiation by delivering miR-92b-3p to MC3T3-E1 cells. miR-92b-3p inhibitor was transfected into the Clino Exos to block miR-92b-3p through electroporation. Then, we administered the modified exosomes to MC3T3-E1 cells. The findings demonstrated that in comparison to the NC-inhibitor group, exosomes carrying miR-92b-3p inhibitor elevated ALP, Osx, Runx2, and Ocn gene expression (Figure 5A). Additionally, the protein levels of Osx, Runx2, and Ocn, ALP activity, and ALP staining demonstrated similar trends (Figure 5B–D). Taken together, all of these results supported that miR-92b-3p was transferred into MC3T3-E1 cells by Clino Exos and was essential for exosome-mediated inhibition of osteogenic differentiation under mechanical unloading.

### 3.6. ELK4 Is a Direct Target of miR-92b-3p and Is Responsible for miR-92b-3p-Mediated Suppression of Osteogenic Differentiation in MC3T3-E1 Cells

**Figure 6:**
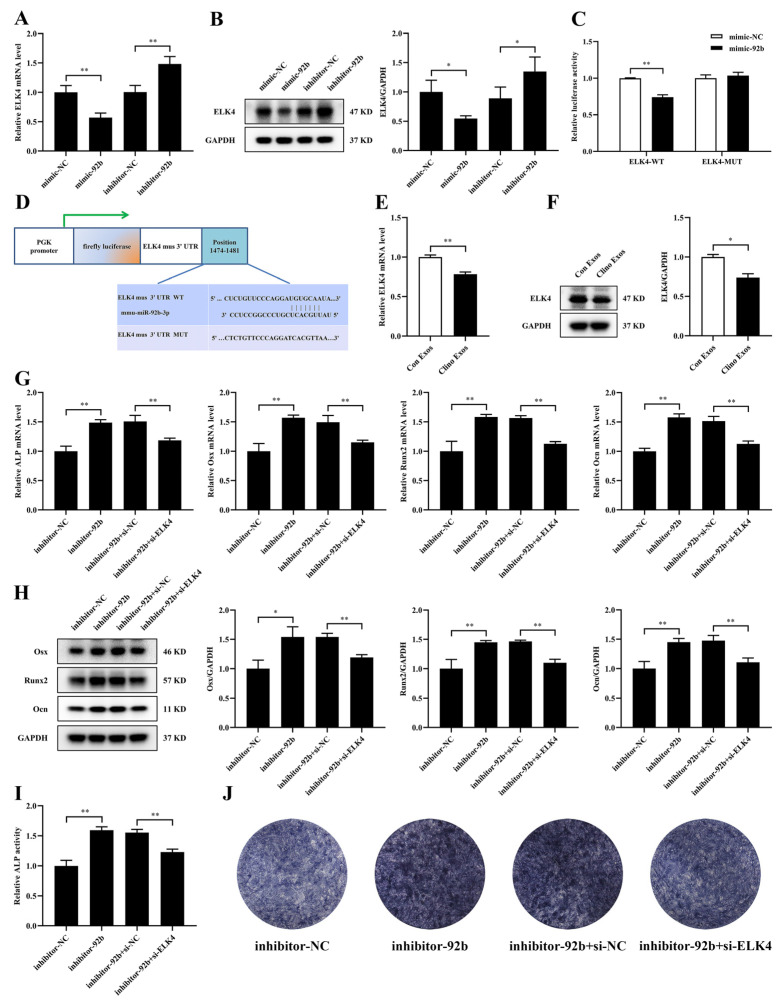
ELK4 is a direct target of miR-92b-3p and is responsible for miR-92b-3p-mediated suppression of osteogenic differentiation in MC3T3-E1 cells. (**A**) qRT–PCR analysis of ELK4 mRNA expression in MC3T3-E1 cells after transfection of mimic-92b-3p, inhibitor-92b-3p, or the corresponding control (*n* = 3). (**B**) Western blotting analysis of the protein expression of ELK4 in MC3T3-E1 cells (*n* = 3). (**C**) The relative luciferase activities of the ELK4 WT and MUT reporters were assessed after 293T cells were treated for 48 h with mimic-92b-3p and the equivalent controls (*n* = 3). (**D**) Schematic representation of the luciferase reporters containing ELK4 3′-UTR WT or MUT sequences. (**E**) mRNA levels of ELK4 analyzed by qRT–PCR in MC3T3-E1 cells treated with Con Exos/Clino Exos (200 μg/mL) (*n* = 3). (**F**) Protein levels of ELK4 analyzed by Western blotting (*n* = 3). (**G**) qRT–PCR analysis of ALP, Osx, Runx2, and Ocn in MC3T3-E1 cells after the co-transfection of inhibitor-92b-3p, si-ELK4 and their negative controls in MC3T3-E1 cells (*n* = 3). (**H**) Western blotting analysis of Osx, Runx2, and Ocn expression in MC3T3-E1 cells (*n* = 3). (**I**) ALP activity analysis in MC3T3-E1 cells (*n* = 3). (**J**) Representative images of ALP staining in MC3T3-E1 cells (*n* = 3). * *p* < 0.05, ** *p* < 0.01 vs. control.

To investigate the molecular mechanism underlying miR-92b-3p-mediated osteoblast differentiation, TargetScan, miRWalk, and miRDB were utilized to predict the potential target genes of miR-92b-3p. According to these analyses, we focused on ELK4, which has been revealed to be involved in signaling pathways associated with osteogenic differentiation [22]. After the overexpression or knockdown of miR-92b-3p, we detected the mRNA and protein expression levels of ELK4 by qRT–PCR and Western blotting. Consequently, we observed that mimic-92b-3p significantly decreased the mRNA and protein level of ELK4, while inhibitor-92b-3p increased them (Figure 6A,B). Next, we performed luciferase reporters containing either the wild-type ELK4 3′UTR (WT) or the ELK4 3′UTR with mutated miR-92b-3p-binding sites (MUT). The luciferase reporter assays illustrated that the ELK4 3′UTR WT luciferase reporter activity was remarkably downregulated by mimic-92b-3p, but no significant changes were found in the ELK4 3′UTR MUT luciferase reporter activity in 293T cells (Figure 6C,D). The results suggested that ELK4 was a direct target of miR-92b-3p.

After MC3T3-E1 cells were incubated with Clino Exos, the mRNA level of ELK4 was reduced (Figure 6E). In addition, the protein level of ELK4 was also downregulated in MC3T3-E1 cells treated with Clino Exos (Figure 6F). To further explore whether the inhibition of miR-92b-3p on osteogenic differentiation depends on ELK4 in MC3T3-E1 cells, we co-transfected inhibitor-92b-3p and siRNA-ELK4 or the corresponding negative controls into MC3T3-E1 cells. siRNA-ELK4 partially suppressed the increase induced by inhibitor-92b-3p in the ALP, Osx, Runx2, and Ocn mRNA expression (Figure 6G). Moreover, the inhibitor-92b-3p-induced promotion of Osx, Runx2, and Ocn protein levels was significantly attenuated by siRNA-ELK4 (Figure 6H). Furthermore, ALP activity and ALP staining demonstrated consistent trends (Figure 6I,J). Therefore, these results illustrated that ELK4 participated in the inhibitory effect of exosomal miR-92b-3p produced by MVECs under mechanical unloading on osteogenic differentiation.

## 4. Discussion

**Figure 7:**
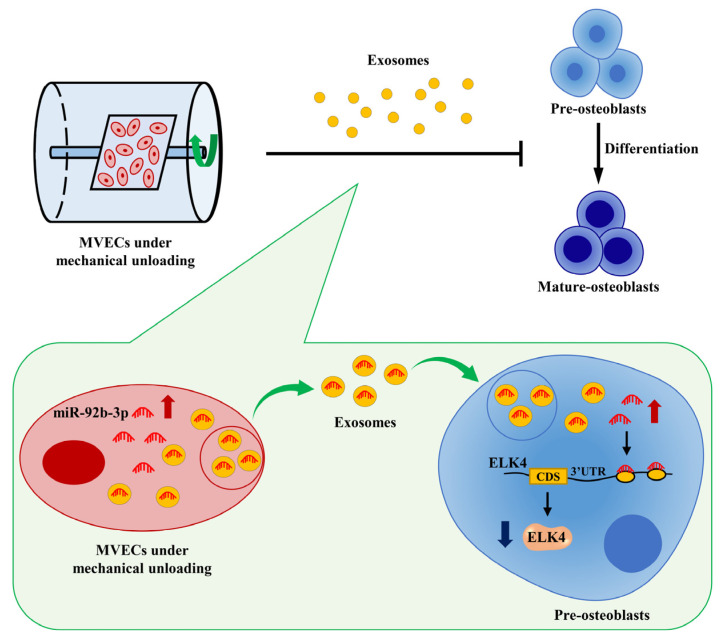
A schematic diagram illustrating the molecular mechanisms of which exosomes derived from MVECs cultured under mechanical unloading regulate osteogenic differentiation. miR-92b-3p expression was increased in MVEC-secreted exosomes after mechanical unloading, resulting in the upregulation of miR-92b-3p expression in MC3T3-E1 cells cocultured with Clino Exos. ELK4, the direct target of miR-92b-3p, is decreased in MC3T3-E1 cells treated with Clino Exos, thus inhibiting osteogenic differentiation. The blue arrow represents inhibition, and the red arrow represents promotion.

The weightless environment in space markedly disrupts the balance between bone formation and bone resorption, causing sustained load-bearing bone loss in astronauts and even disuse osteoporosis [3]. Previous studies have shown that substantial bone loss during unloading in mice was accompanied by a reduction in type H veins in the bone, implying that the skeletal capillaries are involved in bone homeostasis [23]. The endothelium and bone cells interact with one another, and this intercellular communication is essential for maintaining the homeostasis of the bone [10]. It was reported that mechanical unloading affected this intercellular communication, causing reduced ALP activity and decreased calcium deposition [10]. However, little information is available addressing the specific regulation of osteoblasts by MVECs under mechanical unloading. Osteogenic differentiation is a process marked by sequential expression of osteoblast-specific genes (ALP, Osx, Runx2, Ocn, etc.). Therefore, these genes can be used as markers of osteogenic differentiation, and their expression levels can be used to evaluate the degree of osteoblast differentiation [24]. Runx2 is an important regulator of osteogenic differentiation and Runx2 knockdown can cause severe osteogenic dysplasia throughout the skeleton. Moreover, Runx2 regulates the expression of genes such as OPN, BSP, Ocn, and RANKL, thus involving in different stages of osteogenic differentiation [25]. Osx is specifically expressed in all developing bones. Osx is a downstream molecule of Runx2 that affects osteogenic differentiation by inhibiting the Wnt signaling pathway, and Osx knockdown can cause severe impairment of osteogenic differentiation [26]. ALP regulates bone matrix maturation and can be used as an early marker of osteogenic differentiation [27]. Ocn is an important regulatory molecule in bone matrix mineralization and can be used as a late marker of osteogenic differentiation [27,28]. In this work, we revealed that exosomes generated from MVECs under mechanical unloading are involved in the bone loss induced by weightlessness, at least partially through the attenuation of osteogenic differentiation. Mechanistic studies indicated that the elevated miR-92b-3p in the Clino Exos acted as a key factor in exosome-modified suppression of osteoblast differentiation partially by decreasing the mRNA and protein expression of ELK4 (Figure 7). Therefore, our research was the first to confirm that mechanical unloading resulted in miR-92b-3p upregulation in exosomes of MVECs, which reduced osteogenic differentiation via exosomes delivered to MC3T3-E1 cells.

Exosomes contain a variety of bioactive substances and transport them to target cells and are thus involved in intercellular signal transduction [29]. Moreover, exosomes transport lipids, proteins, and nucleic acids into target cells by fusing directly with the cytosolic membrane of target cells, being absorbed through endocytosis, and recognizing specific receptors on the target cells’ surface to regulate the physiological activities of target cells [30,31]. Jia et al. reported that exosomes produced by BMSCs facilitated fracture healing in vivo by the translocation of miR-25 to control Runx2 ubiquitination degradation by SMURF1 [17]. Furthermore, exosomes derived from osteoclasts transferred miR-214-3p into osteoblasts to suppress osteoblast activity and decrease bone formation in mice [19,20]. Interestingly, investigations have indicated that exosomes generated from bEnd.3 cells have better bone targeting ability and biocompatibility than exosomes generated from BMSCs and MC3T3-E1 cells [32]. Therefore, we verified for the first time that exosomes derived from MVECs under mechanical unloading repressed osteoblast differentiation by coculturing MC3T3-E1 cells with Con Exos or Clino Exos. We further hypothesized that the inhibitory effect of MVEC-derived exosomes in mechanical unloading could rely on miRNA delivery. Among the candidates suggested by our small RNA sequencing results, we chose to focus on miR-92b-3p because its expression was significantly upregulated. Consistently, in comparison with the respective control groups, we further confirmed that the expression of miR-92b-3p was elevated in MVECs under clinorotation unloading, in the exosomes that they produced, and in MC3T3-E1 cells cultured with Clino Exos. Therefore, these results suggested that MVEC-derived exosomes can successfully deliver miR-92b-3p to osteoblasts.

There is debate over miR-92b-3p’s function in tumors. miR-92b-3p is regarded as a carcinogenic factor in several cancers, including colorectal, gastric, bladder, and prostate cancer [33,34]. Moreover, miR-92b-3p exhibits anticancer effects in glioma, pancreatic cancer, and esophageal squamous cell carcinoma [35,36]. Additionally, miR-92b-3p suppression induced migration, invasion, and EMT through increasing COL1A2 expression in human lens epithelial cells [37]. Interestingly, miR-92b-3p shares a seed sequence with miR-363-3p. A previous study proposed that osteoporotic samples showed the greatest level of miR-363-3p expression [38]. miR-363-3p promoted osteoclast-specific gene expression but reduced the expression of osteoblast-specific markers through targeting PTEN to boost PI3K/AKT signals [38]. It has also been suggested that high expression of miR-363-3p attenuated osteoblast differentiation and enhanced adipogenic differentiation and senescence by targeting TRAF3 in BMSCs [39]. Nevertheless, there have been few reports of miR-92b-3p’s role in osteoblast differentiation. Intriguingly, Chen et al. proposed that miR-92b-3p impeded vascular calcification through decreasing KLF4 and Runx2 expression in vascular smooth muscle cells [40]. Gain- and loss-of-function experiments utilizing miR-92b-3p mimic or inhibitor, respectively, proved that miR-92b-3p impaired osteogenic differentiation. Furthermore, we observed that treatment of electroporated Clino Exos with miR-92b-3p inhibitor alleviated the suppression of osteogenic differentiation in MC3T3-E1 cells.

ETS-like transcription factor 4 (ELK4) belongs to the ternary complex factor subfamily of E twenty-six domain transcription genes [41]. ELK4 participates in a series of mechanisms, such as cell proliferation, differentiation, apoptosis, and inflammation [41,42,43]. ELK4 is regarded as an oncogenic gene in several cancers, such as melanoma, prostate, gastric, and lung cancers [44]. Intriguingly, recent studies have discovered that the expression of ELK4, which exerts effects on BMP receptor signaling pathway, was upregulated in stem cells from human exfoliated deciduous teeth [22]. Moreover, the BMP receptor signaling pathway may strongly influence the transformation of human exfoliated deciduous tooth stem cells into osteogenic/odontogenic cells [22]. In this study, we reported that the expression of ELK4 was reduced in MC3T3-E1 cells treated with Clino Exos and that ELK4 was a direct target molecule of miR-92b-3p. We further confirmed that siRNA-ELK4 partially attenuated inhibitor-92b-3p-promoted osteoblast differentiation. Taken together, the findings of this work indicated that MVECs under mechanical unloading retarded osteoblastic differentiation by delivering exosomal miR-92b-3p and downregulating ELK4. Notably, our study mainly focuses on the effects of exosomal miRNAs in osteoblastic differentiation. However, numerous studies have reported that endothelial cells in the skeletal microenvironment secret plenty of vascular endocrine factors to regulate bone growth and homeostasis, such as Hedgehog, Notch, WNT, BMP, FGF, IGF, PDGF, etc. [7,45,46]. Apart from miRNAs, exosomes secreted by MVECs under mechanical unloading may contain these cytokines and undergo changes in expression, which in turn may be involved in modulating the inhibition of osteogenic differentiation. This also requires in-depth research and will help to elucidate comprehensively the molecular mechanisms of exosome regulation of disuse osteoporosis.

## 5. Conclusions

In conclusion, our study first revealed that exosomes secreted from MVECs cultured under mechanical unloading attenuated osteogenic differentiation and miR-92b-3p was upregulated in exosomes of MVECs cultured under mechanical unloading. Furthermore, miR-92b-3p was delivered into MC3T3-E1 cells via exosomes leading to the inhibition of osteogenic differentiation, which partly depended on ELK4 regulation. Our research uncovered an exosomal miR-92b-3p/ELK4 signaling pathway that regulated osteoblast differentiation, suggesting that exosomes may be a promising therapeutic strategy for disuse osteoporosis.

## Data Availability

Not applicable.

## Supplementary Materials

The following supporting information can be downloaded at: https://www.mdpi.com/article/10.3390/jpm12122030/s1, Table S1: Primer sequences used for qRT–PCR, Table S2: RNA oligo sequences for transfection. Figure S1. Clino Exos attenuate osteoblast differentiation of MC3T3-E1 cells in a dose-dependent manner. Figure S2. The effects of Clino Exos and Con Exos on osteogenic differentiation in MC3T3-E1 cells compared with no exosomes. Figure S3. ELK4 promotes osteogenic differentiation in MC3T3-E1 cells.

## References

[1] Carina V., Della Bella E., Costa V., Bellavia D., Veronesi F., Cepollaro S., Fini M., Giavaresi G. (2020). Bone’s Response to Mechanical Loading in Aging and Osteoporosis: Molecular Mechanisms. Calcif. Tissue Res..

[2] Hsieh Y.-F., Turner C.H. (2001). Effects of Loading Frequency on Mechanically Induced Bone Formation. J. Bone Miner. Res..

[3] Lang T., Leblanc A., Evans H., Lu Y., Genant H., Yu A. (2004). Cortical and Trabecular Bone Mineral Loss from the Spine and Hip in Long-Duration Spaceflight. J. Bone Miner. Res..

[4] Leblanc A., Schneider V., Shackelford L., West S., Oganov V., Bakulin A., Voronin L. (2000). Bone mineral and lean tissue loss after long duration space flight. J. Musculoskelet. Neuronal. Interact..

[5] Sambandam Y., Baird K.L., Stroebel M., Kowal E., Balasubramanian S., Reddy S.V. (2016). Microgravity Induction of TRAIL Expression in Preosteoclast Cells Enhances Osteoclast Differentiation. Sci. Rep..

[6] Blaber E.A., Dvorochkin N., Lee C., Alwood J.S., Yousuf R., Pianetta P., Globus R.K., Burns B.P., Almeida E.A.C. (2013). Microgravity Induces Pelvic Bone Loss through Osteoclastic Activity, Osteocytic Osteolysis, and Osteoblastic Cell Cycle Inhibition by CDKN1a/p21. PLoS ONE.

[7] Zhu S., Bennett S., Kuek V., Xiang C., Xu H., Rosen V., Xu J. (2020). Endothelial cells produce angiocrine factors to regulate bone and cartilage via versatile mechanisms. Theranostics.

[8] Laranjeira M.S., Fernandes M.H., Monteiro F.J. (2012). Reciprocal induction of human dermal microvascular endothelial cells and human mesenchymal stem cells: Time-dependent profile in a co-culture system. Cell Prolif..

[9] Ribeiro V., Garcia M., Oliveira R., Gomes P.S., Colaço B., Fernandes M.H. (2013). Bisphosphonates induce the osteogenic gene expression in co-cultured human endothelial and mesenchymal stem cells. J. Cell. Mol. Med..

[10] Cazzaniga A., Castiglioni S., Maier J.A.M. (2014). Conditioned Media from Microvascular Endothelial Cells Cultured in Simulated Microgravity Inhibit Osteoblast Activity. BioMed Res. Int..

[11] Kourembanas S. (2015). Exosomes: Vehicles of Intercellular Signaling, Biomarkers, and Vectors of Cell Therapy. Annu. Rev. Physiol..

[12] Kalluri R., LeBleu V.S. (2020). The biology, function, and biomedical applications of exosomes. Science.

[13] Fazi F., Nervi C. (2008). MicroRNA: Basic mechanisms and transcriptional regulatory networks for cell fate determination. Cardiovasc. Res..

[14] Hu Z., Wang Y., Sun Z., Wang H., Zhou H., Zhang L., Zhang S., Cao X. (2015). miRNA-132-3p inhibits osteoblast differentiation by targeting Ep300 in simulated microgravity. Sci. Rep..

[15] Wang Y., Wang K., Hu Z., Zhou H., Zhang L., Wang H., Li G., Zhang S., Cao X., Shi F. (2018). MicroRNA-139-3p regulates osteoblast differentiation and apoptosis by targeting ELK1 and interacting with long noncoding RNA ODSM. Cell Death Dis..

[16] Wang K., Wang Y., Hu Z., Zhang L., Li G., Dang L., Tan Y., Cao X., Shi F., Zhang S. (2020). Bone-targeted lncRNA OGRU alleviates unloading-induced bone loss via miR-320-3p/Hoxa10 axis. Cell Death Dis..

[17] Jiang Y., Zhang J., Li Z., Jia G. (2020). Bone Marrow Mesenchymal Stem Cell-Derived Exosomal miR-25 Regulates the Ubiquitination and Degradation of Runx2 by SMURF1 to Promote Fracture Healing in Mice. Front. Med..

[18] Cui Y., Luan J., Li H., Zhou X., Han J. (2015). Exosomes derived from mineralizing osteoblasts promote ST2 cell osteogenic differentiation by alteration of microRNA expression. FEBS Lett..

[19] Sun W., Zhao C., Li Y., Wang L., Nie G., Peng J., Wang A., Zhang P., Tian W., Li Q. (2016). Osteoclast-derived microRNA-containing exosomes selectively inhibit osteoblast activity. Cell Discov..

[20] Li D., Liu J., Guo B., Liang C., Dang L., Lu C., He X., Cheung H.Y.-S., Xu L., Lu C. (2016). Osteoclast-derived exosomal miR-214-3p inhibits osteoblastic bone formation. Nat. Commun..

[21] Xu L., Zhang X., Li G., Zhang L., Zhang S., Shi F., Hu Z. (2022). Inhibition of SIRT1 by miR-138-5p provides a mechanism for inhibiting osteoblast proliferation and promoting apoptosis under simulated microgravity. Life Sci. Space Res..

[22] Hara K., Yamada Y., Nakamura S., Umemura E., Ito K., Ueda M. (2011). Potential Characteristics of Stem Cells from Human Exfoliated Deciduous Teeth Compared with Bone Marrow–derived Mesenchymal Stem Cells for Mineralized Tissue-forming Cell Biology. J. Endod..

[23] Liang S., Ling S., Du R., Li Y., Liu C., Shi J., Gao J., Sun W., Li J., Zhong G. (2020). The coupling of reduced type H vessels with unloading-induced bone loss and the protection role of Panax quinquefolium saponin in the male mice. Bone.

[24] Ducy P., Karsenty G. (1995). Two distinct osteoblast-specific cis-acting elements control expression of a mouse osteocalcin gene. Mol. Cell. Biol..

[25] Ducy P., Zhang R., Geoffroy V., Ridall A.L., Karsenty G. (1997). Osf2/Cbfa1: A Transcriptional Activator of Osteoblast Differentiation. Cell.

[26] Nakashima K., Zhou X., Kunkel G., Zhang Z., Deng J.M., Behringer R.R., de Crombrugghe B. (2002). The Novel Zinc Finger-Containing Transcription Factor Osterix Is Required for Osteoblast Differentiation and Bone Formation. Cell.

[27] Zhang J., Zhang W., Dai J., Wang X., Shen S.G. (2019). Overexpression of Dlx2 enhances osteogenic differentiation of BMSCs and MC3T3-E1 cells via direct upregulation of Osteocalcin and Alp. Int. J. Oral. Sci..

[28] Gundberg C.M. (2000). Biochemical Markers of Bone Formation. Clin. Lab. Med..

[29] Pegtel D.M., Gould S.J. (2019). Exosomes. Annu. Rev. Biochem..

[30] Xie X., Xiong Y., Panayi A.C., Hu L., Zhou W., Xue H., Lin Z., Chen L., Yan C., Mi B. (2020). Exosomes as a Novel Approach to Reverse Osteoporosis: A Review of the Literature. Front. Bioeng. Biotechnol..

[31] Kobayashi M., Sawada K., Miyamoto M., Shimizu A., Yamamoto M., Kinose Y., Nakamura K., Kawano M., Kodama M., Hashimoto K. (2020). Exploring the potential of engineered exosomes as delivery systems for tumor-suppressor microRNA replacement therapy in ovarian cancer. Biochem. Biophys. Res. Commun..

[32] Song H., Li X., Zhao Z., Qian J., Wang Y., Cui J., Weng W., Cao L., Chen X., Hu Y. (2019). Reversal of Osteoporotic Activity by Endothelial Cell-Secreted Bone Targeting and Biocompatible Exosomes. Nano Lett..

[33] Wang G., Cheng B., Jia R., Tan B., Liu W. (2020). Altered expression of microRNA-92b-3p predicts survival outcomes of patients with prostate cancer and functions as an oncogene in tumor progression. Oncol. Lett..

[34] Huang J., Wang B., Hui K., Zeng J., Fan J., Wang X., Hsieh J.-T., He D., Wu K. (2016). miR-92b targets DAB2IP to promote EMT in bladder cancer migration and invasion. Oncol. Rep..

[35] Long M., Zhan M., Xu S., Yang R., Chen W., Zhang S., Shi Y., Yongheng S., Mohan M., Liu Q. (2017). miR-92b-3p acts as a tumor suppressor by targeting Gabra3 in pancreatic cancer. Mol. Cancer.

[36] Xu T., Wang H., Jiang M., Yan Y., Li W., Xu H., Huang Q., Lu Y., Chen J. (2017). The E3 ubiquitin ligase CHIP/miR-92b/PTEN regulatory network contributes to tumorigenesis of glioblastoma. Am. J. Cancer Res..

[37] Huang P., Hu Y., Duan Y. (2022). TGF-β2-induced circ-PRDM5 regulates migration, invasion, and EMT through the miR-92b-3p/COL1A2 pathway in human lens epithelial cells. Histochem. J..

[38] Li M., Luo R., Yang W., Zhou Z., Li C. (2019). miR-363-3p is activated by MYB and regulates osteoporosis pathogenesis via PTEN/PI3K/AKT signaling pathway. Vitr. Cell. Dev. Biol. Anim..

[39] Wang D., Cai G., Wang H., He J. (2020). TRAF3, a Target of MicroRNA-363-3p, Suppresses Senescence and Regulates the Balance between Osteoblastic and Adipocytic Differentiation of Rat Bone Marrow-Derived Mesenchymal Stem Cells. Stem Cells Dev..

[40] Chen C., Li Y., Lu H., Liu K., Jiang W., Zhang Z., Qin X. (2022). Curcumin attenuates vascular calcification via the exosomal miR-92b-3p/KLF4 axis. Exp. Biol. Med..

[41] Zheng L., Xu H., Di Y., Chen L., Liu J., Kang L., Gao L. (2021). ELK4 promotes the development of gastric cancer by inducing M2 polarization of macrophages through regulation of the KDM5A-PJA2-KSR1 axis. J. Transl. Med..

[42] Wernert N., Shaikhibrahim Z., Lindstrot A., Langer B., Buettner R. (2011). Differential expression of ETS family members in prostate cancer tissues and androgen-sensitive and insensitive prostate cancer cell lines. Int. J. Mol. Med..

[43] Day B.W., Stringer B.W., Spanevello M.D., Charmsaz S., Jamieson P.R., Ensbey K.S., Carter J.C., Cox J.M., Ellis V.J., Brown C.L. (2011). ELK4 neutralization sensitizes glioblastoma to apoptosis through downregulation of the anti-apoptotic protein Mcl-1. Neuro-Oncology.

[44] Yang W., Gao K., Qian Y., Huang Y., Xiang Q., Chen C., Chen Q., Wang Y., Fang F., He Q. (2022). A novel tRNA-derived fragment AS-tDR-007333 promotes the malignancy of NSCLC via the HSPB1/MED29 and ELK4/MED29 axes. J. Hematol. Oncol..

[45] Zhu S., Yao F., Qiu H., Zhang G., Xu H., Xu J. (2017). Coupling factors and exosomal packaging microRNAs involved in the regulation of bone remodelling. Biol. Rev..

[46] Ramasamy S.K., Kusumbe A.P., Itkin T., Gur-Cohen S., Lapidot T., Adams R.H. (2016). Regulation of Hematopoiesis and Osteogenesis by Blood Vessel–Derived Signals. Annu. Rev. Cell Dev. Biol..

