# Supplementary material for: Exosomes from Microvascular Endothelial Cells under Mechanical Unloading Inhibit Osteogenic Differentiation via miR-92b-3p/ELK4 Axis
Source: J Pers Med. 2022 Dec 8;12(12):2030. doi: 10.3390/jpm12122030 (PMC9785449; doi:10.3390/jpm12122030)
Supplement: Supplementary file 1 [file jpm-12-02030-s001.zip › jpm-2027690-supplementary.pdf]

Supplementary Materials for

Exosomes from microvascular endothelial cells under mechanical unloading inhibit osteogenic differentiation via miR-92b-3p/ELK4 axis

Xiaoyan Zhang†, Lijun Zhang†, Liqun Xu, Gaozhi Li, Ke Wang, Tong Xue, Quan Sun, Hao Tang, Xinsheng Cao, Zebing Hu, Shu Zhang*, Fei Shi*

*To whom correspondence should be addressed: Shu Zhang at shuzhang@fmmu.edu.cn; Fei Shi at shifei719@fmmu.edu.cn.

Table S1 Primer sequences used for qRT–PCR

Gene	Sequence（5'~3'）
**U6-F**	CTCGCTTCGGCAGCACA
**U6-R**	AACGCTTCACGAATTTGCGT
**mmu-miR-31-3p**	TCGTGCTATGCCAACATATTGCCATC
**mmu-miR-92b-3p**	ATATTGCACTCGTCCCGGCC
**mmu-miR-19b-3p**	CGTGTGCAAATCCATGCAAAACTGA
**mmu-miR-199a-5p**	AATGCTCCCAGTGTTCAGACTA
**mmu-miR-199a-3p**	GCACTGACAGTAGTCTGCACATTG
**mmu-miR-19a-3p**	CGCCTGTGCAAATCTATGCAAAACTGA
**mmu-miR-350-3p**	CCGCTTCACAAAGCCCATACACTTTC
**mmu-miR-223-3p**	AAGAGCGTTGTCAGTTTGTCAA
**mRQ 3' Primer**	TGGTGTCGTGGAGTCG
**GAPDH-F**	TGTGTCCGTCGTGGATCTGA
**GAPDH-R**	TTGCTGTTGAAGTCGCAGGAG
**ELK4-F**	ACTGACGGTAGGCAGGATTGAGG
**ELK4-R**	GTCATTGCGACTAGAGGTCTTGGC
**Osx-F**	AAGAGGTTCACCCGCTCTGA
**Osx-R**	TGATGTTTGCTCAAGTGGTCG
**Runx2-F**	GAACCAAGAAGGCACAGACAGA

**Runx2-R**	GGCGGGACACCTACTCTCATAC
**ALP-F**	GCAGTATGAATTGAATCGGAACAAC
**ALP-R**	ATGGCCTGGTCCATCTCCAC
**Ocn-F**	GACCGCCTACAAACGCATCTA
**Ocn-R**	CAGAGAGAGAGGACAGGGAGGA

Table S2 RNA oligo sequences for transfection

**Name**	**Sequence (5' - 3')**
**Mimic-NC sense**	UUCUCCGAACGUGUCACGUTT
**Mimic-NC antisense**	ACGUGACACGUUCGGAGAATT
**Inhibitor-NC**	CAGUACUUUUGUGUAGUACAA
**Mimic-92b-3p sense**	UAUUGCACUCGUCCCGGCCUCC
**Mimic-92b-3p antisense**	AGGCCGGGACGAGUGCAAUAUU
**Inhibitor-92b-3p**	GGAGGCCGGGACGAGUGCAAUA
**Negative control sense**	UUCUCCGAACGUGUCACGUTT
**Negative control antisense**	ACGUGACACGUUCGGAGAATT
**siRNA-ELK4 sense**	GUCGCAAUGACUACAUACATT
**siRNA-ELK4 antisense**	UGUAUGUAGUCAUUGCGACTT

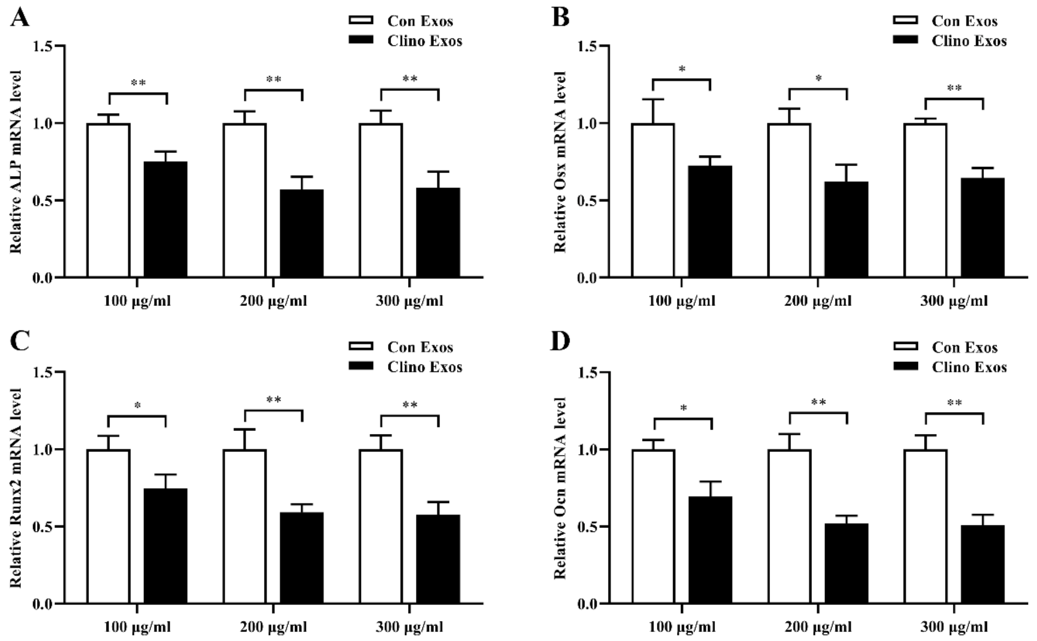

Figure S1. Clino Exos attenuate osteoblast differentiation of MC3T3-E1 cells in a dose-dependent manner. (A) The mRNA level of ALP in MC3T3-E1 cells by qRT–PCR (n=3). (B) The mRNA level of Osx in MC3T3-E1 cells by qRT–PCR (n=3). (C) The mRNA level of Runx2 in MC3T3-E1 cells by qRT–PCR (n=3). (D) The mRNA level of Ocn in MC3T3-E1 cells by qRT–PCR (n=3). *P < 0.05, **P < 0.01 vs. control.

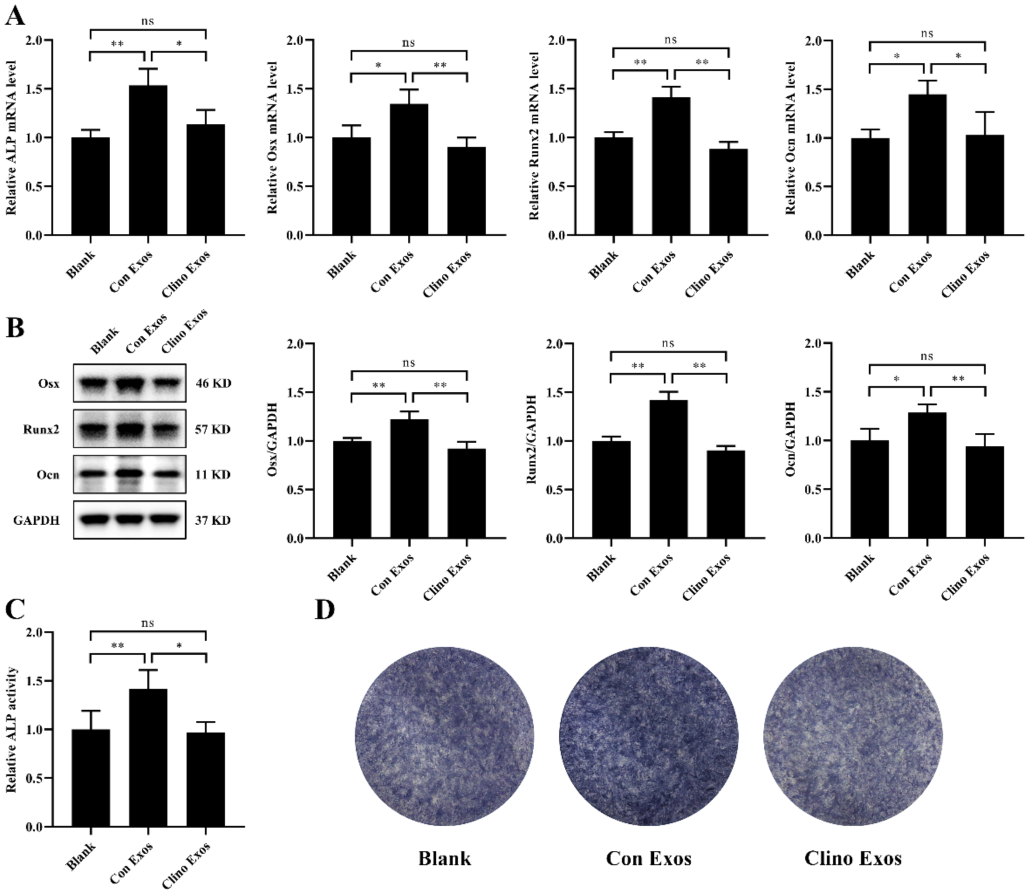

Figure S2. The effects of Clino Exos and Con Exos on osteogenic differentiation in MC3T3-E1 cells compared with no exosomes. (A) The mRNA level of ALP, Osx, Runx2, and Ocn in MC3T3-E1 cells treated with PBS/Con Exos/Clino Exos (200 μg/ml) by qRT–PCR (n=3). (B) The protein level of Osx, Runx2, and Ocn in MC3T3-E1 cells treated with PBS/Con Exos/Clino Exos (200 μg/ml) by Western blotting (n=3). (C) Relative ALP activity analysis in MC3T3-E1 cells treated with PBS/Con Exos/Clino Exos (200 μg/ml) (n=3). (D) Representative images of ALP staining in MC3T3-E1 cells with PBS/Con Exos/Clino Exos (200 μg/ml) after 7 days osteogenic induction (n=3). *P < 0.05, **P < 0.01 vs. control.

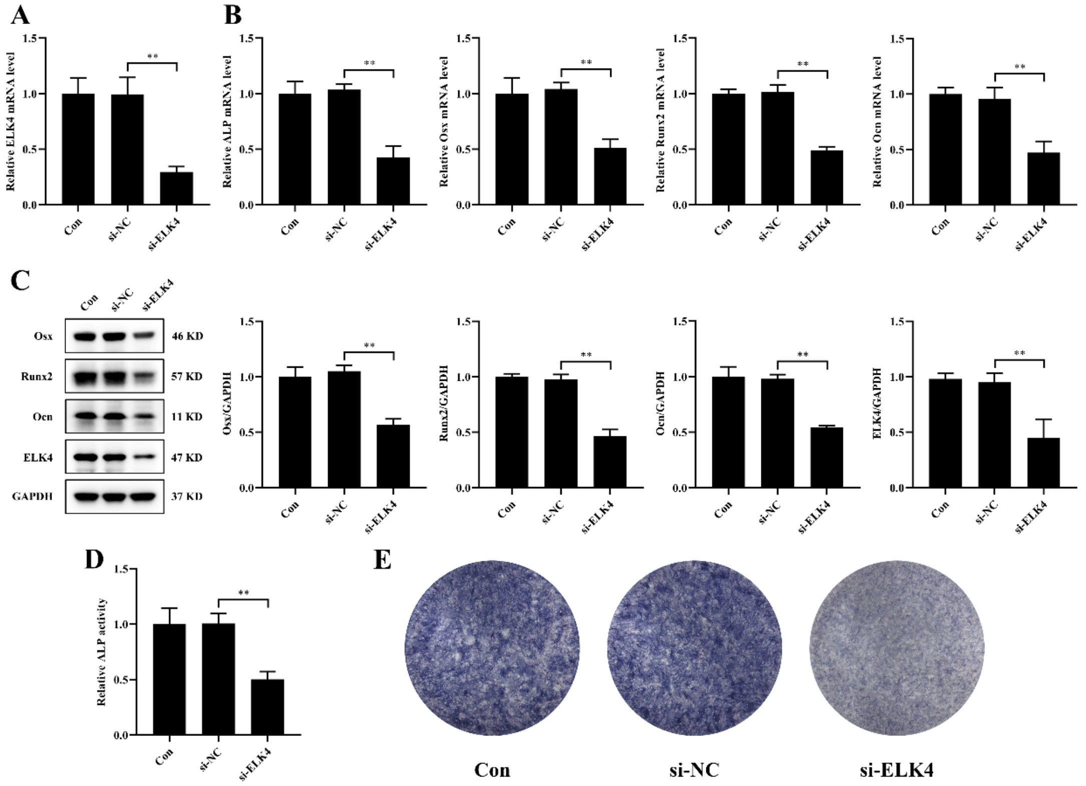

Figure S3. ELK4 promotes osteogenic differentiation in MC3T3-E1 cells. (A) The mRNA level of ELK4 in MC3T3-E1 cells by qRT–PCR after transfection of si-ELK4 or the negative control (n=3). (B) The mRNA level of ALP, Osx, Runx2, and Ocn in MC3T3-E1 cells by qRT–PCR after transfection of si-ELK4 or the negative control (n=3). (C) The protein level of Osx, Runx2, Ocn, and ELK4 in MC3T3-E1 cells by Western blotting (n=3). (D) Relative ALP activity analysis in MC3T3-E1 cells. (E) Representative images of ALP staining in MC3T3-E1 cells (n=3). **$P < 0.01$ vs. control.
